# Augmented frontal cortex diacylglycerol levels in Parkinson’s disease and Lewy Body Disease

**DOI:** 10.1371/journal.pone.0191815

**Published:** 2018-03-07

**Authors:** Paul L. Wood, Soumya Tippireddy, Joshua Feriante, Randall L. Woltjer

**Affiliations:** 1 Metabolomics Unit, College of Veterinary Medicine, Lincoln Memorial University, Cumberland Gap Pkwy., Harrogate, TN, United States of America; 2 DeBusk College of Osteopathic Medicine, Lincoln Memorial University, Cumberland Gap Pkwy., Harrogate, TN, United States of America; 3 Department of Neurology, Oregon Health Science University and Portland VA Medical Center, Portland, OR, United States of America; McGill University, CANADA

## Abstract

**Background:**

Research from our laboratory, and that of other investigators, has demonstrated augmented levels of diacylglycerols (DAG) in the frontal cortex and plasma of subjects with Alzheimer’s disease (AD) and Mild Cognitive Impairment (MCI). We have extended these observations to investigate the frontal cortex of subjects with Parkinson’s disease (PD) and Lewy Body Disease (LBD), with and without coexisting pathologic features of AD.

**Methods/Principal findings:**

Utilizing a high-resolution mass spectrometry analytical platform, we clearly demonstrate that DAG levels are significantly increased in the frontal cortex of subjects with PD, LBD with intermediate neocortical AD neuropathology, and in LBD with established neocortical AD neuropathology. In the case of the PD cohort, increases in cortical DAG levels were detected in cases with no neocortical pathology but were greater in subjects with neocortical pathology. These data suggest that DAG changes occur early in the disease processes and are amplified as cortical dysfunction becomes more established.

**Conclusions:**

These findings suggest that altered DAG synthesis/metabolism is a common feature of neurodegenerative diseases, characterized by proteinopathy, that ultimately result in cognitive deficits. With regard to the mechanism responsible for these biochemical alterations, selective decrements in cortical levels of phosphatidylcholines in LBD and PD suggest that augmented degradation and/or decreased synthesis of these structural glycerophospholipids may contribute to increases in the pool size of free DAGs. The observed augmentation of DAG levels may be phospholipase-driven since neuroinflammation is a consistent feature of all disease cohorts. If this conclusion can be validated it would support utilizing DAG levels as a biomarker of the early disease process and the investigation of early intervention with anti-inflammatory agents.

## Introduction

Extensive lipidomics evaluations of subjects with Alzheimer’s disease (AD) have demonstrated alterations in the levels of glycerophospholipids, sphingolipids, and diacylglycerols (DAG) in plasma and brain [1–3]. The most unique and consistent observation of these lipidomics studies is the elevation of DAG levels in AD frontal cortex [4–7] and mixed dementia temporal cortex [8]. Similarly DAG levels are augmented in the plasma of AD [9–11] and MCI [11] subjects, and appear to occur in a subset of patients encompassing 25 to 30% of MCI subjects and 40 to 45% of AD subjects [11].

Elevated DAG levels have also been reported from a lipidomics analysis of the visual cortex from 10 sporadic PD cases [12]. As is the case with MCI and AD, PD is characterized by a proteinopathy and usually proceeds to cognitive dysfunction [13]. While AD pathology involves amyloid plaques and neurofibrillary tangles, PD and Lewy body disease (LBD) are characterized by alpha-synuclein protein aggregates that form Lewy bodies [14–15]. As has been observed in AD, there are dramatic decrements in cortical cholinergic pathways [16–18] and cortical thinning [19–20], which presumably reflect processes of cognitive dysfunction in PD and LBD. However, post-mortem studies have demonstrated that many subjects have multiple proteinopathies [21] and that in the case of synucleinopathies, as in AD, there is a great deal of phenotypic heterogeneity [22–23] including various coexisting pathologic features of AD. Therefore, we extended our lipidomics evaluations of altered DAG metabolism in proteinopathies to PD and LBD subjects. Specifically, the cases were classified into 4 groups based on extensive neuropathological evaluations. These included a control group, a LBD group with early to intermediate-stage AD pathology (LBD-I-AD), a LBD group with established neocortical AD pathology (LBD-AD), and a PD group in which our investigation did not detect neocortical Lewy bodies or neurofibrillary tangles of AD.

## Materials and methods

### Study samples

Frontal cortex samples, bio banked at the Oregon Brain Bank, were from volunteer subjects who were evaluated at Oregon Health and Science University. IRB approval was from the OHSU Research Integrity Office (approval IRB1623). Postmortem samples were fully anonymized at the time of accession and informed consent was obtained before autopsy from the next of kin for all areas of research. Patients themselves were expired and did not provide consent. Frontal lobe tissue was flash frozen and stored at -80° C for biochemical studies. Tissue for morphologic evaluation was taken from all cortical lobes bilaterally, the anterior cingulate gyrus, hippocampus, amygdala, bilateral striatum and thalamus, midbrain, pons, medulla, and cerebellum as previously described [24]. In brief, sections from these regions were stained with hematoxalin and eosin with Luxol fast blue myelin stain. A modified Bielschowski silver impregnation method [25] as well as immunohistochemical staining for β-amyloid (Biolegend, San Diego, 4G8 antibody to β-amyloid amino acids 17–24) was used to identify diffuse and neuritic plaques in frontal and parietal cortices, hippocampus, basal ganglia, and cerebellum. Tau immunohistochemistry was performed on the hippocampus, frontal cortex, and occipital cortex using PHF1 antibody (a gift from Dr. Peter Davies, Albert Einstein College of Medicine, Manhasset, NY). The presence of Lewy bodies was determined by anti-alpha-synuclein immunohistochemistry using LB509 antibody (Covance, Princeton, NJ) performed on sections of midbrain, amygdala, hippocampus, and frontal cortex. Brain tissue was evaluated by a neuropathologist for features of neurodegenerative disease, which were identified and staged as previously reported and according to the NACC’s uniform data collection. Table 1 summarizes the patient demographics and neuropathologic features.

**Table 1:** Patient demographic and neuropathologic features of control, Lewy body disease with intermediate Alzheimer’s pathology (LBD-I-AD), Lewy body disease with Alzheimer’s pathology (LBD-AD), and Parkinson’s disease (PD) subjects. PMI (post-mortem interval).

Parameter	Controls	LBD-I-AD	LBD-AD	PD
**N**	43	20	29	15
**Age (Yrs ± SD)** **[Range]**	67.1 ± 8.4 [55–83]	80.9 ± 10.5 [67–90]	74.3 ± 8.8 [61–93]	82.8 ± 9.5 [70–100]
**Gender (% Males)**	60.5	65.0	62.1	73.3
**PMI (Hrs ± SD)** **[Range]**	25.3 ± 16.9 [3–72]	14.0 ± 15.1 [3–41]	20.9 ± 27.18 [2–120]	22.2 ± 25.4 [2–96]
**Neocortical Plaques**	Absent	Present	Present	Variable
**Neocortical Neurofibrillary Tangles**	Absent	III-IV	V-VI	Absent
**Midbrain Lewy bodies**	Absent	Present	Present	Present
**Neocortical Lewy bodies**	Absent	Present	Present	Absent

### Lipid extraction and analysis

Lipids were extracted from 20 to 40 mg of brain tissue with methyl-tert-butyl ether and methanol (1 mL methanol, 1 mL distilled water, and 2 mL methyl-tert-butyl ether) containing stabled isotope standards as described previously [5–7, 9, 11, 25–26]. Extracts were dried by centrifugal vacuum evaporation and dissolved in isopropanol: methanol: chloroform (4:2:1) containing 15 mM ammonium acetate. Constant infusion lipidomics (10 μL per min) were performed utilizing high-resolution (140,000; 0.3 to 3 ppm mass error) data acquisition on an orbitrap mass spectrometer (Thermo Q Exactive). Washes (500 μL) with methanol followed by hexane/ethyl acetate: chloroform (3:2:2), between samples, were used to minimize ghost effects.

In positive ion ESI, the cations of choline plasmalogens, phosphatidylcholines, sphingomyelins, ceramides, galactosylceramides, lactosylceramides, and the ammonium adducts of diacylglycerols (DAG) were quantitated. The cation of bromocriptine was used to monitor for potential mass axis drift. In negative ion ESI, the anions of ethanolamine plasmalogens, sulfatides, phosphatidylserines, phosphatidylethanolamines, phosphatidylglycerols, phosphatidylinositols, and lysophosphatidylglycerols were monitored. The anion of bromocriptine was used to monitor for potential mass axis drift.

The masses and the MS^2^ validation data for critical lipids are summarized in Table 2.

**Table 2:** Exact masses and MS^2^ data for reported lipids.

**Lipid**	**Exact**	**Calc. Cation**	**ppm**	**Product**	**Energy**	**Calc. Cation**	**ppm**
Cer d18:1/18:0	565.5434	566.5507	1.7	d18:1 base	25	264.26913	3.1
Gal-Cer d18:1/24:1	809.6744	810.6817	2.5	d18:1 base	20	264.26913	3.1
				[MH-Gal]	15	630.61832	0.36
Lac-Cer d18:1/18:1	887.6334	888.6407	2.1	d18:1 base	20	264.26913	3.4
				[MH-Lac]	15	546.5245	1.8
**Lipid**	**Exact**	**Calc. Anion**	**ppm**	**Product**	**Energy**	**Calc. Anion**	**ppm**
Sulfatide d18:1/24:0	891.6469	890.6397	0.5	HSO4	50	96.9595	1.1
Sulfatide d18:1/24:1	889.6313	888.6240	1.7	HSO4	50	96.9595	0.9
Sulfatide d18:1/25:0	905.6626	904.6583	1.8	HSO4	50	96.9595	1.2
Sulfatide d18:1/26:1	917.6625	916.6553	1.8	HSO4	50	96.9595	1.0
**Lipid**	**Exact**	**Calc. Cation**	**ppm**	**Product**	**Energy**	**Calc. Cation**	**ppm**
DAG 34:1 (16:0/18:1)	594.5223	612.5567	2.6	[M-sn1]	25	339.2894	0.88
				[M-sn2]	25	313.2737	1.2
DAG 36:1 (18:0/18:1)	622.5536	640.5880	2.1	[M-sn1]	25	339.2894	0.87
				[M-sn2]	25	341.3050	0.58
DAG 36:2 (18:1/18:1)	620.5379	638.5724	2.1	[M-sn1]	25	339.2894	0.92
				[M-sn2]	25	339.2894	0.92
DAG 36:4 (16:0/20:4)	616.5067	634.5411	3.0	[M-sn1]	25	361.2738	1.3
				[M-sn2]	25	313.2738	1.5
DAG 38:6 (16:0/22:6)	640.5066	658.5410	2.7	[M-sn1]	25	385.2737	0.77
				[M-sn2]	25	313.2737	1.5
PlsC 32:0	717.5672	718.5745	2.1	Phosphocholine	25	184.0474	1.2
PlsC 34:0	745.5981	746.6088	1.8	Phosphocholine	25	184.0474	1.1
PtdC 34:5	751.5152	752.5225	2.7	Phosphocholine	25	184.0474	1.0
PtdC 36:5	779.5465	780.5538	2.6	Phosphocholine	25	184.0474	0.8
PtdC 38:5	807.5778	808.5851	3.4	Phosphocholine	25	184.0474	0.9
PtdC 38:6	805.5621	806.5694	2.8	Phosphocholine	25	184.0474	1.1
SM 22:1	784.6458	785.6531	2.6	Phosphocholine	25	184.0474	1.1
SM 24:0	814.6928	815.7001	2.8	Phosphocholine	25	184.0474	1.2
SM 24:2	810.6615	811.6688	2.5	Phosphocholine	25	184.0474	0.7
SM 26:1	840.7085	841.7157	2.7	Phosphocholine	25	184.0474	1.1
**Lipid**	**Exact**	**Calc. Anion**	**ppm**	**Product**	**Energy**	**Calc. Antion**	**Ppm**
PlsE 38:3	753.5672	752.5599	1.6	sn1 18:1	25	281.2486	2.2
				sn2 20:2	25	307.2642	0.58
PlsE 38:6	747.5203	746.5165	1.8	sn1 16:0	25	255.2329	0.96
				sn2 22:6	25	327.2329	0.53
PlsE 40:6	775.5516	774.5473	1.5	sn1 18:0	25	283.2642	2.0
				sn2 20:6	25	327.2329	0.52
LPA 16:0	410.2433	409.2360	0.67	P-Glycerol	25	152.9953	2.1
PtdG 32:0	722.5098	721.5025	1.7	sn1 16:0	25	255.2329	0.65
				P-Glycerol	25	152.9953	2.2
LPG 16:0	484.2801	483.2728	0.18	16:0	25	255.2329	0.72
				P-Glycerol	25	152.9953	2.1
PtdS 36:1	789.5520	788.5447	1.3	sn1 18:0	25	283.2642	2.2
				sn2 18:1	25	281.2486	2.0
PtdS 36:2	787.5364	786.5291	1.4	sn1 18:1	25	281.2486	2.0
				sn2 18:1	25	281.2486	2.0
PtdS 38:3	813.5520	812.5447	2.7	sn1 18:0	25	283.2642	2.1
				sn2 20:2	25	307.2642	0.61

### Statistical analysis

R values (ratio of endogenous lipid peak area to the peak area of an appropriate internal standard) were calculated. R values were corrected for the wet weight of the tissue analyzed. Individual R values are presented in S1 Table. Data are presented as mean ± SEM. Data were analyzed with ANOVA, followed by the Dunnett’s test to compare groups.

## Results

### Diacylglycerols (DAG)

**Fig 1:**
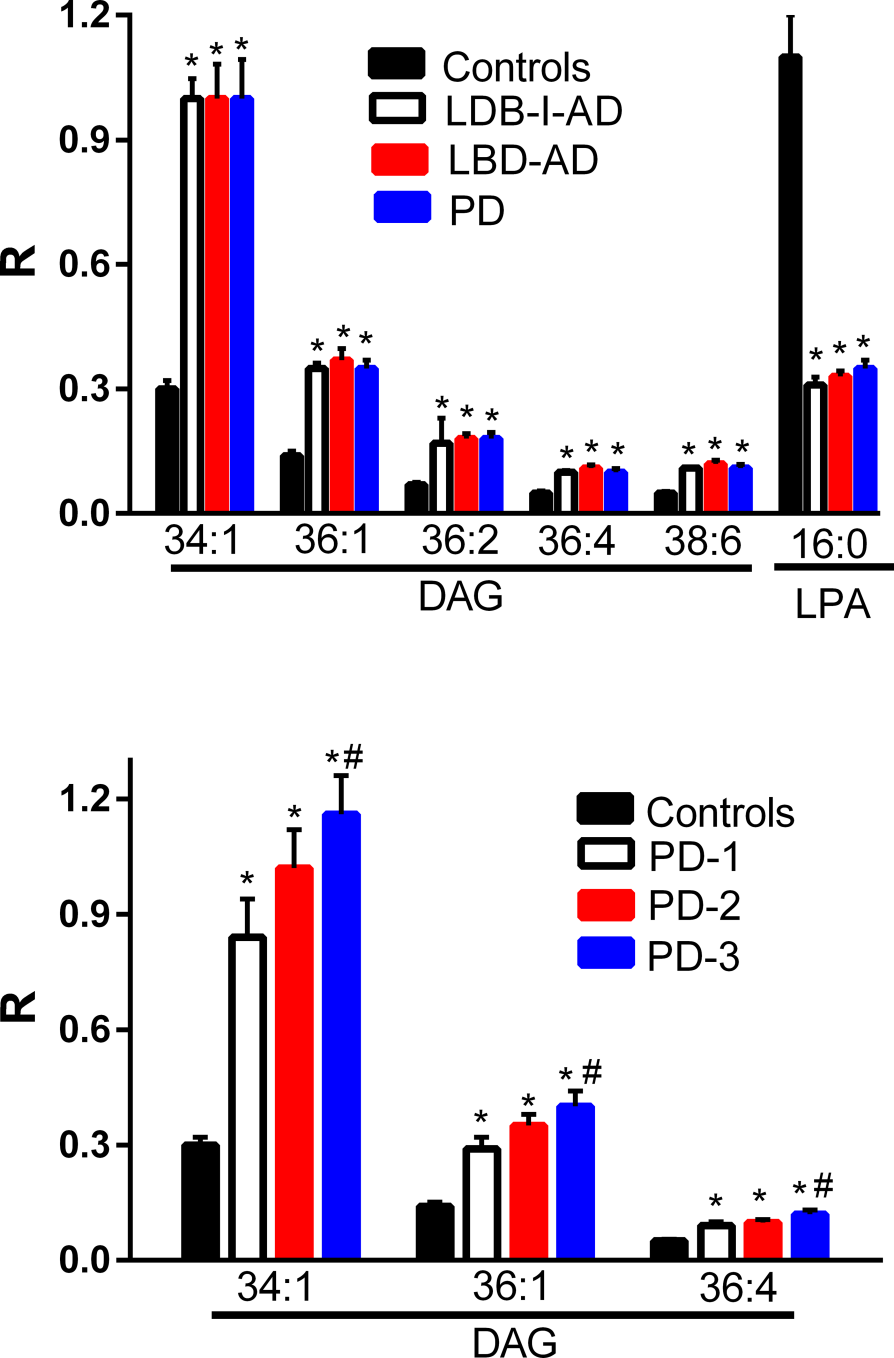
Frontal cortex levels of diacylglycerols (DAG) and lysophosphatidic acid 16:0 (LPA) in control, Lewy body disease with intermediate Alzheimer’s disease (LBD-I-AD), Lewy body disease with Alzheimer’s disease (LBD-AD), and Parkinson’s disease (PD) tissues. R = ratio of the peak area of the endogenous lipid to the peak area of the internal standard (mean ±SEM). Analysis of PD subgroups demonstrated that DAGs were augmented in the cortex of PD subjects with no neocortical pathology (PD-1, N = 5), subjects with sparse neocortical neuritic plaques (PD-2, N = 5), and subjects with moderate to frequent neocortical neuritic plaques (PD-3, N = 5). *, p < 0.01 vs. controls; #, p < 0.05 for PD-2 vs. PD3.

DAGs with both monounsaturated and polyunsaturated fatty acid side chains were significantly increased in the frontal cortex of the LBD-I-AD, LBD-AD, and PD groups (Fig 1). These data are similar to observations with AD frontal cortex [4–5]. Analysis of subgroups in the PD cohort revealed that DAGs were elevated (Fig 1) in subjects with no neocortical pathology (PD-1), subjects with sparse neocortical neuritic plaques (PD-2), and subjects with moderate to frequent neocortical neuritic plaques (PD-3). In addition there were greater DAG elevations in the cohort with the most severe cortical neuropathology (Fig 1).

In contrast, the levels of lysophosphatidic acid 16:0, a precursor of DAGs, were significantly decreased in all 3 pathological groups (Fig 1). PD subgroup analysis did not reveal any significant differences in LPA levels between the PD-1, PD-2, or PD-3 subgroups.

### Plasmalogens

**Fig 2:**
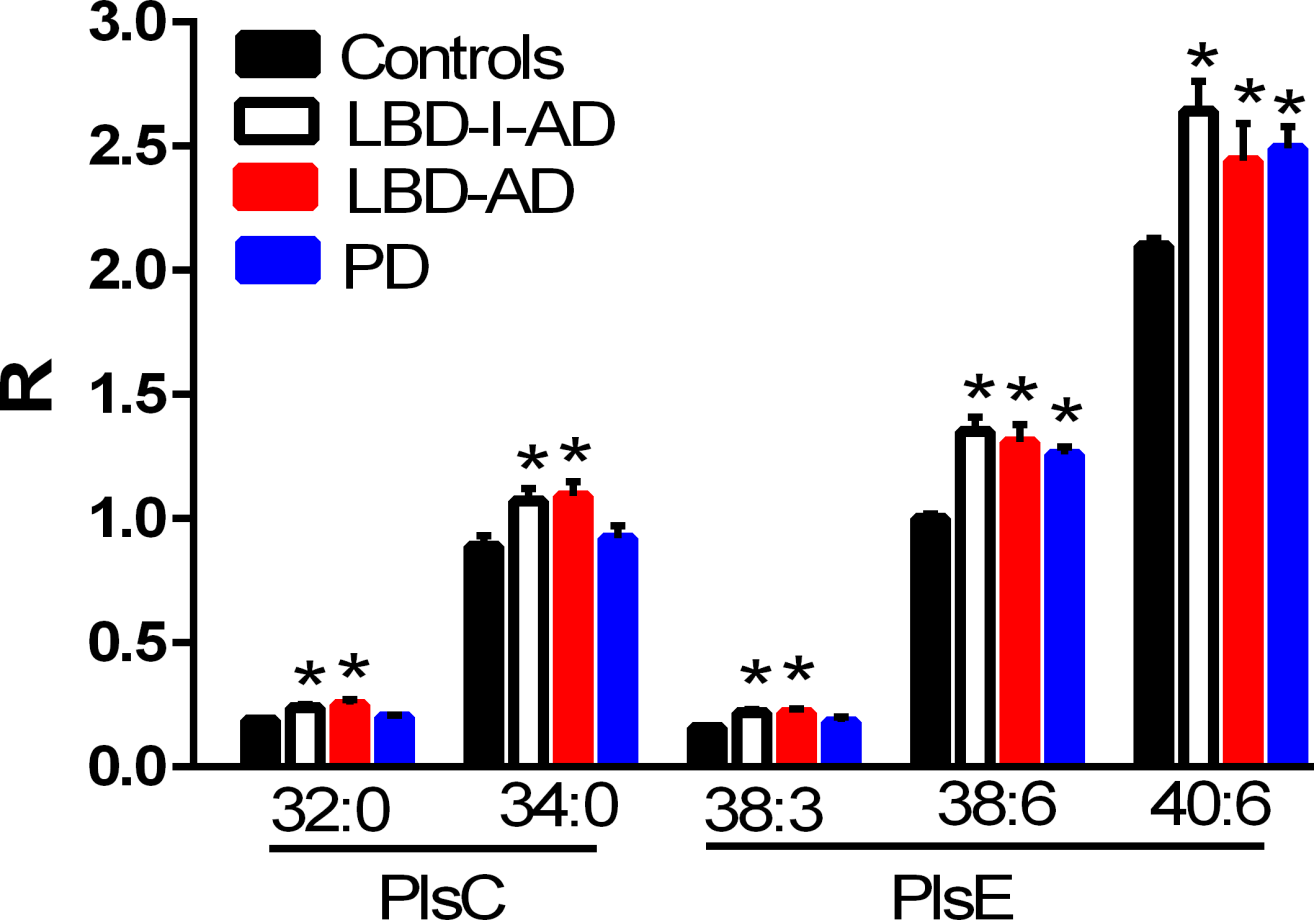
Frontal cortex levels of plasmalogens in control, Lewy body disease with intermediate Alzheimer’s disease (LBD-I-AD), Lewy body disease with Alzheimer’s disease (LBD-AD), and Parkinson’s disease (PD) tissues. PlsC, choline plasmalogens; PlsE, ethanolamine plasmalogen. R = ratio of the peak area of the endogenous plasmalogen to the peak area of the internal standard (mean ±SEM). *, p < 0.05 vs controls.

Both choline and ethanolamine plasmalogens were found to be increased in the frontal cortex of the LBD-I-AD and LBD-AD groups (Fig 2). Ethanolamine plasmalogens also tended to be augmented in PD frontal cortex (Fig 2). These data contrast with AD where plasmalogens tend to be decreased in the frontal cortex [4–5]. PD subgroup analysis did not reveal any significant differences in plasmalogen levels between the PD-1, PD-2, or PD-3 subgroups.

### Sphingolipids

**Fig 3:**
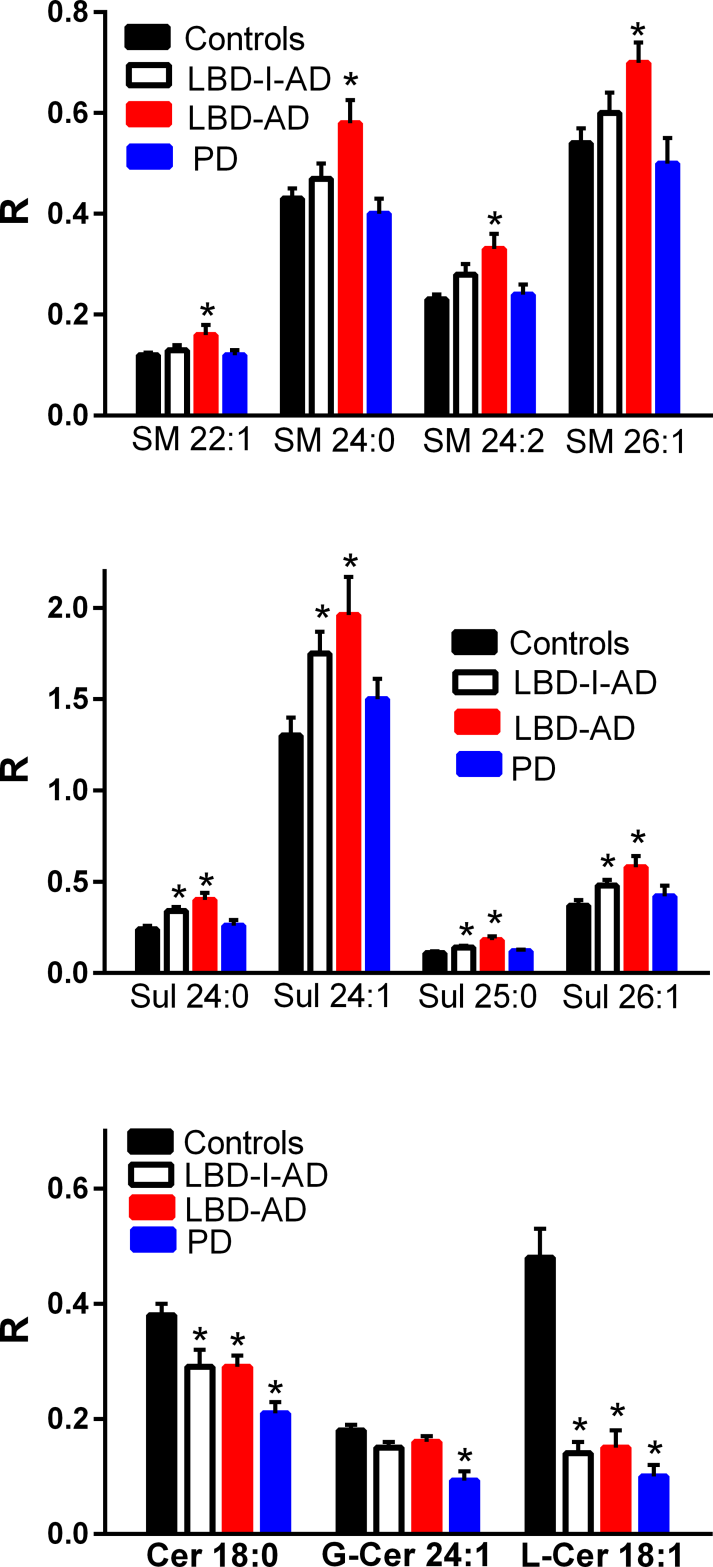
Frontal cortex levels of sphingolipids (SM, sphingomyelin; Sul, sulfatide, Cer, ceramide; G-Cer, galactosyceramide; L-Cer, lactosylceramide) in control, Lewy body disease with intermediate Alzheimer’s disease (LBD-I-AD), Lewy body disease with Alzheimer’s disease (LBD-AD), and Parkinson’s disease (PD) tissues. R = ratio of the peak area of the endogenous sphingolipid to the peak area of the internal standard (mean ±SEM). *, p < 0.05. vs controls.

Several sphingomyelins were elevated in the frontal cortex but exclusively in the LBD-AD group while sulfatides were augmented in the frontal cortex of the LBD-I-AD and LBD-AD cohorts and not in the PD cohort (Fig 3). These data contrast with sulfatide decrements and unaltered sphingomyelin levels in AD cortex [4–5]. Ceramide metabolism also was altered, with decrements in ceramide d18:1 and lactosylceramide in all 3 disease groups while galactosyl/glucosyl-ceramide 24:1 was selectively decreased in the cortex of the PD cohort (Fig 3).

### Phosphatidyl (diacyl) glycerophospholipids

**Fig 4:**
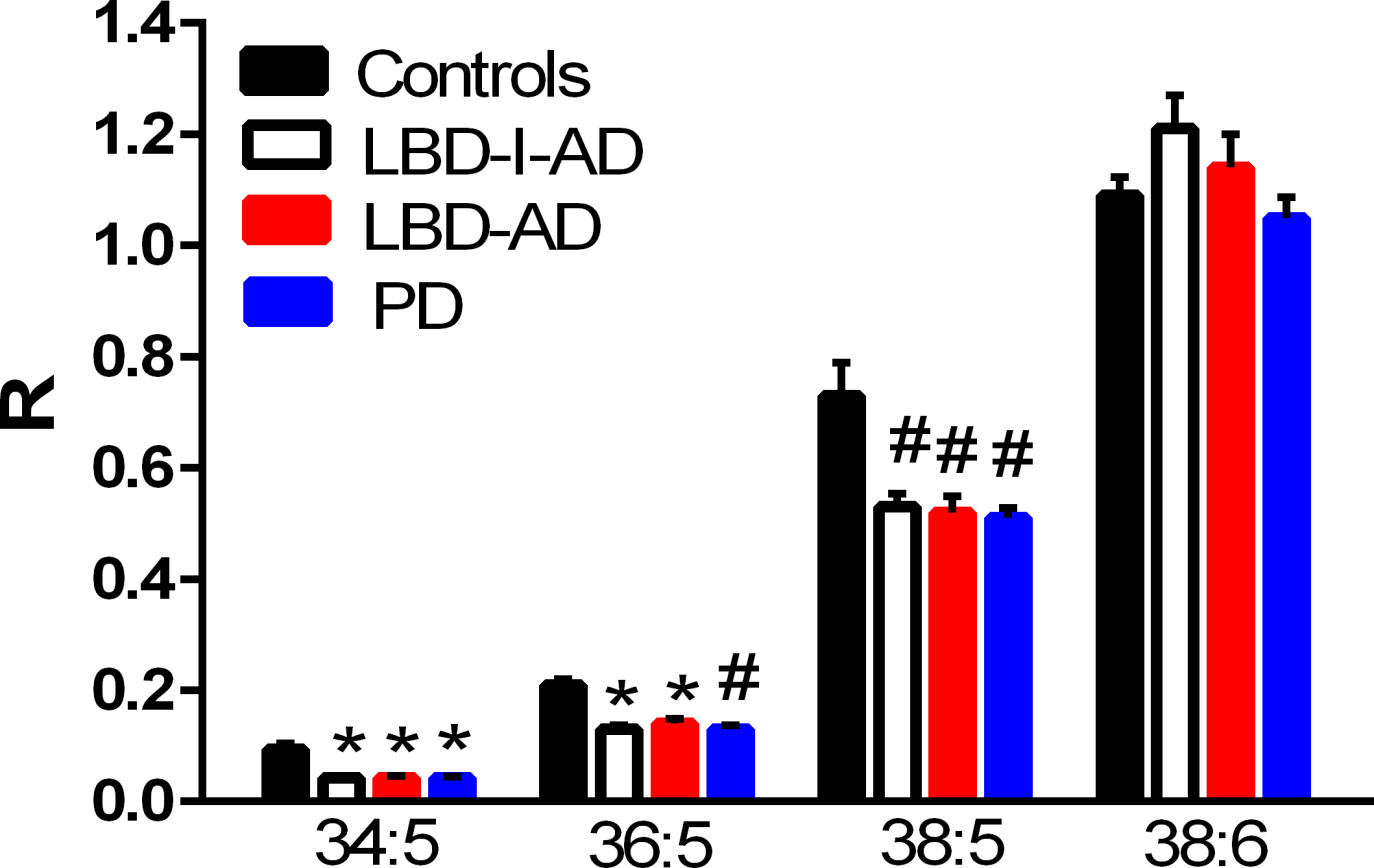
Decreased frontal cortex levels of 20:5-containing phosphatidylcholines (PtdC) in Lewy body disease with intermediate Alzheimer’s disease (LBD-I-AD), Lewy body disease with Alzheimer’s disease (LBD-AD), and Parkinson’s disease (PD) tissues. R = ratio of the peak area of the endogenous PtdC to the peak area of the internal standard (mean ±SEM). *, p < 0.01; #, p < 0.05 vs controls.

**Fig 5:**
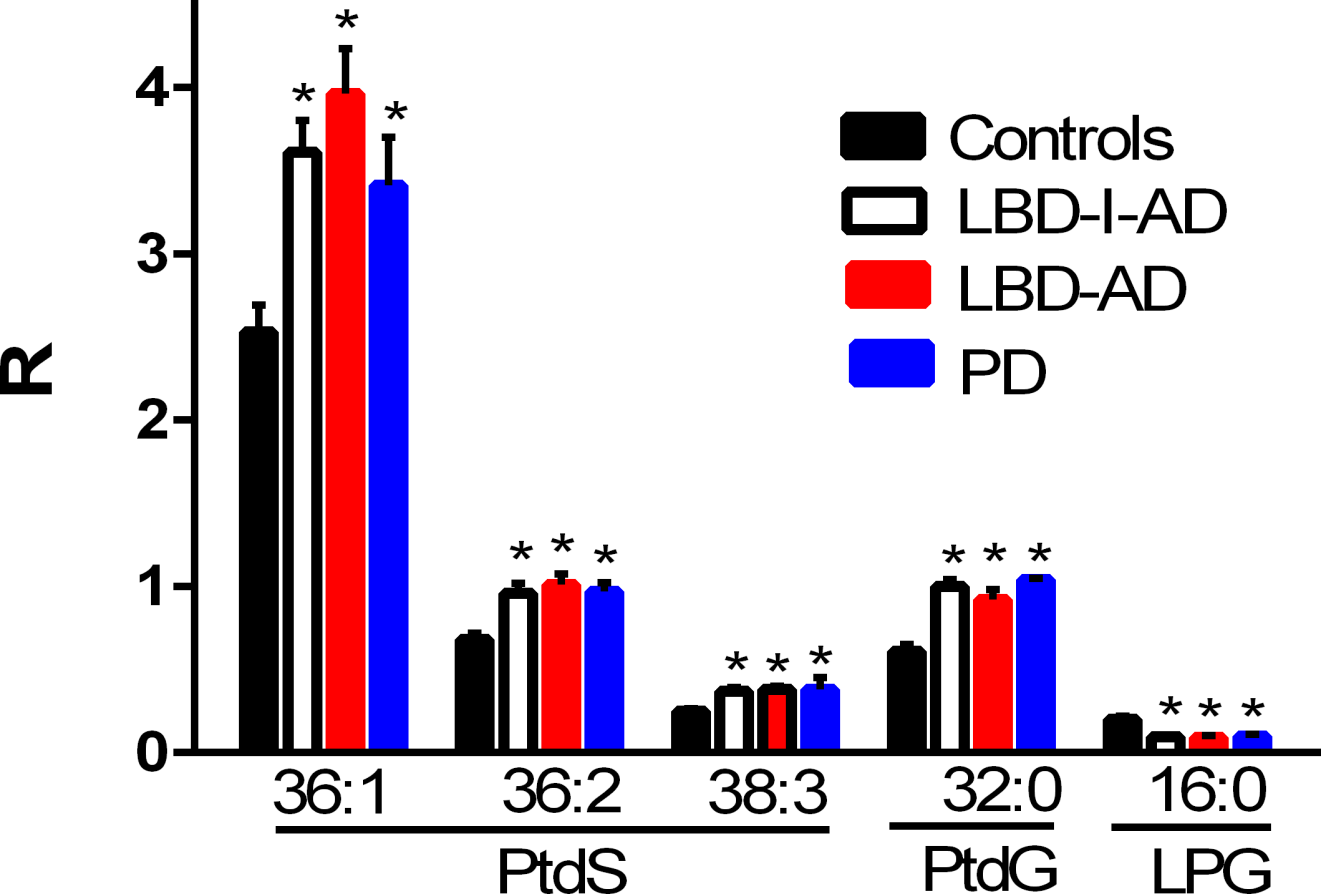
Frontal cortex levels of phosphatidylserines (PtdS), phosphatidylglycerol 32:0 (PtdG), and lysophosphatidylglycerol 16:0 (LPG) in control, Lewy body disease with intermediate Alzheimer’s disease (LBD-I-AD), Lewy body disease with Alzheimer’s disease (LBD-AD), and Parkinson’s disease (PD) tissues. R = ratio of the peak area of the endogenous lipid to the peak area of the internal standard (mean ±SEM). *, p < 0.01 vs controls.

Phosphatidylcholines with 20:5 substitutions were decreased in all 3 neuropathological groups (Fig 4). This included phosphatidylcholines (PC) PC 34:5, PC 36:5, and PC 38:5 (Fig 4). In contrast, phosphatidylserines and phosphatylylglycerols were augmented in the frontal cortex of all 3 neuropathological groups (Fig 5). In concert with augmented phosphatidylglycerol levels, decrements in the levels of lysophosphatidylglycerols were measured (Fig 5). PD subgroup analysis did not reveal any significant differences in diacyl glycerophospholipid levels between the PD-1, PD-2, or PD-3 subgroups.

## Discussion

With the emergence of cognitive dysfunction in elderly patients, the practice of medicine is complicated in that mixed pathologies are present in many individuals. In addition, there is a variable penetrance of these pathologies resulting in a large phenotypic variation [27–28]. This heterogeneity limits the ability of physicians to provide a clinical diagnosis that would allow for earlier therapeutic intervention. In addition the co-morbidities of multiple ongoing pathologies complicates our ability to define potential points of intervention for new therapeutic strategies.

**Fig 6:**
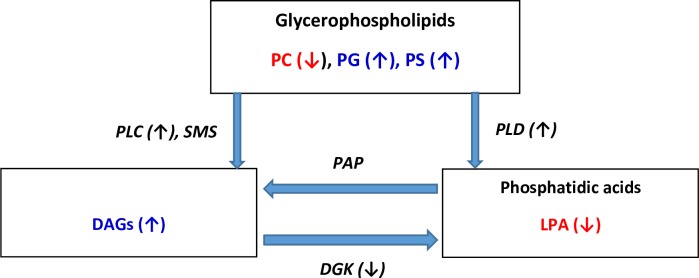
Abbreviated schematic presentation of DAG metabolism. LPA, lysophosphatidic acid; PC, phosphatidylcholine; PG, phosphatidylglycerol; PS, phosphatidylserine; PLC, phospholipase C; PLD, phospholipase D; SMS, sphingomyelin synthase; DGK, diacylglycerol kinase. Red indicates lowered levels and blue indicates increased levels observed in our study.

Disruption of lipid homeostasis appears to be a common biochemical feature of the investigated proteinopathies and may contribute to the neuronal dysfunction responsible for cognitive decline. The observed increases in DAG levels in the frontal cortex of AD, PD, and LBD subjects are of key interest since DAGs are essential for the synthesis of structural glycerophospholipids, for energy metabolism, and act as mediators of signal transduction, including nuclear signaling. DAGs are essential second messengers in the nuclear lipid signaling pathway with levels being tightly controlled by DAG kinase (Fig 6) which terminates DAG signaling via conversion of DAGs to phosphatidic acids [29–30]. Our observations of elevated DAGs and lower levels of lysophosphatidic acid suggest that there may be a dysfunction in the DAG kinase regulation of DAG steady–state levels in proteinopathies. This conjecture may be of import since disruption of DAG kinase function has be speculated to contribute to both neuroinflammation [31] and amyloid deposition [32]. The microRNA, miR-34a, a negative regulator of DAG kinase [33], is up-regulated in frontal cortex in AD [34] suggesting that metabolism of DAGs by DAG kinase may be decreased in AD brain. Unfortunately DAG kinase (10 isoforms) has not been characterized in synucleinopathies.

The other major metabolic route, to regulate DAG levels, is their utilization in the synthesis of diacylglycerophospholipids (Fig 6). In this case, both choline (EC 2.7.8.2) and ethanolamine (EC 2.7.8.1) phosphotransferase have been measured in human brain [35] and in isolated synaptosomes [36], supporting the essential roles of these enzymes in neuronal function. Metabolism of glycerophospholipids to DAGs involves phospholipase C and phospholipase D (PLD) [6, 37]. However, the augmented levels of phosphatidylserines, phosphatidylglycerols and plasmalogens, along with unaltered phosphatidylethanolamine and phosphatidylinositol levels observed in our study, does not suggest that global alterations in these metabolic pathways contribute to the elevated DAG pools. The specific decreases in phosphatidylcholine levels suggests that this glycerophospholipid pool is selectively metabolized to DAGs or that there is a selective decrease in choline phosphotransferase activity in synucleinopathies that results in decreased phosphatidylcholine synthesis.

Other metabolic sources of DAG include *de novo* synthesis from the glycolytic pathway and from sphingolipid metabolism (Fig 6). Since glucose hypometabolism appears to predominate in most neurodegenerative disorders [38] and plasma glucose levels are generally unaltered in AD [6], this is less likely to contribute to the reported increases in plasma and brain DAG levels. With regard to sphingolipid metabolism, the synthesis of sphingomyelins which involves the transfer of a phosphocholine head group to a ceramide (EC 2.7.8.27; sphingomyelin synthase) results in the generation of DAGs [36]. The augmented sphingomyelin levels observed in the LDB-AD group in our study and the prior study of PD [12] suggest that this metabolic pathway also may be a contributing factor to augmented DAG levels in the frontal cortex in late-stage synucleinopathies. This contrasts with studies of AD where sphingomyelin levels were found to be unaltered in the frontal cortex [4–5]. While altered sphingolipid metabolism has been hypothesized to be involved in the induction of PD [39], the inconsistent findings of altered sphingolipid metabolites between laboratories remain to be resolved before firm conclusions can be drawn [12, 40–41].

A role of inflammation in the augmentation of augmented plasma DAGs in MCI and AD, and of neuroinflammation in the augmentation of brain levels of DAGs may be important since inflammation is known to result in induction of PLD [42–44]. In dementias brain microglia presumably are involved in the induction PLD and other neuroinflammatory pathways [45–51]. Similarly, peripheral inflammation may be responsible for elevated levels of DAGs in AD [7, 9, 10, 52] and MCI [7, 9] plasma. In contrast individuals possessing normal cognition until death at greater than 85 years of age did not demonstrate elevations in DAGs [5, 6]. However, at autopsy these subjects demonstrated significant AD neuropathology and have been coined non-demented AD neuropathology (NDAN). These data suggest that neuritic plaques and tangles or Lewy bodies do not initiate the observed alterations in DAG levels but may exacerbate the ongoing biochemical changes. The early detection of neuroinflammation and elevated DAG levels in MCI, AD, PD, and LBD suggest that early anti-inflammatory interventions may ultimately be neuroprotective and block cognitive decline. This conclusion is supported by the observations that individuals utilizing repeated NSAID use have a reduced incidence of AD while established AD patients do not benefit for NSAID therapy [52, 53].

**Fig 7:**
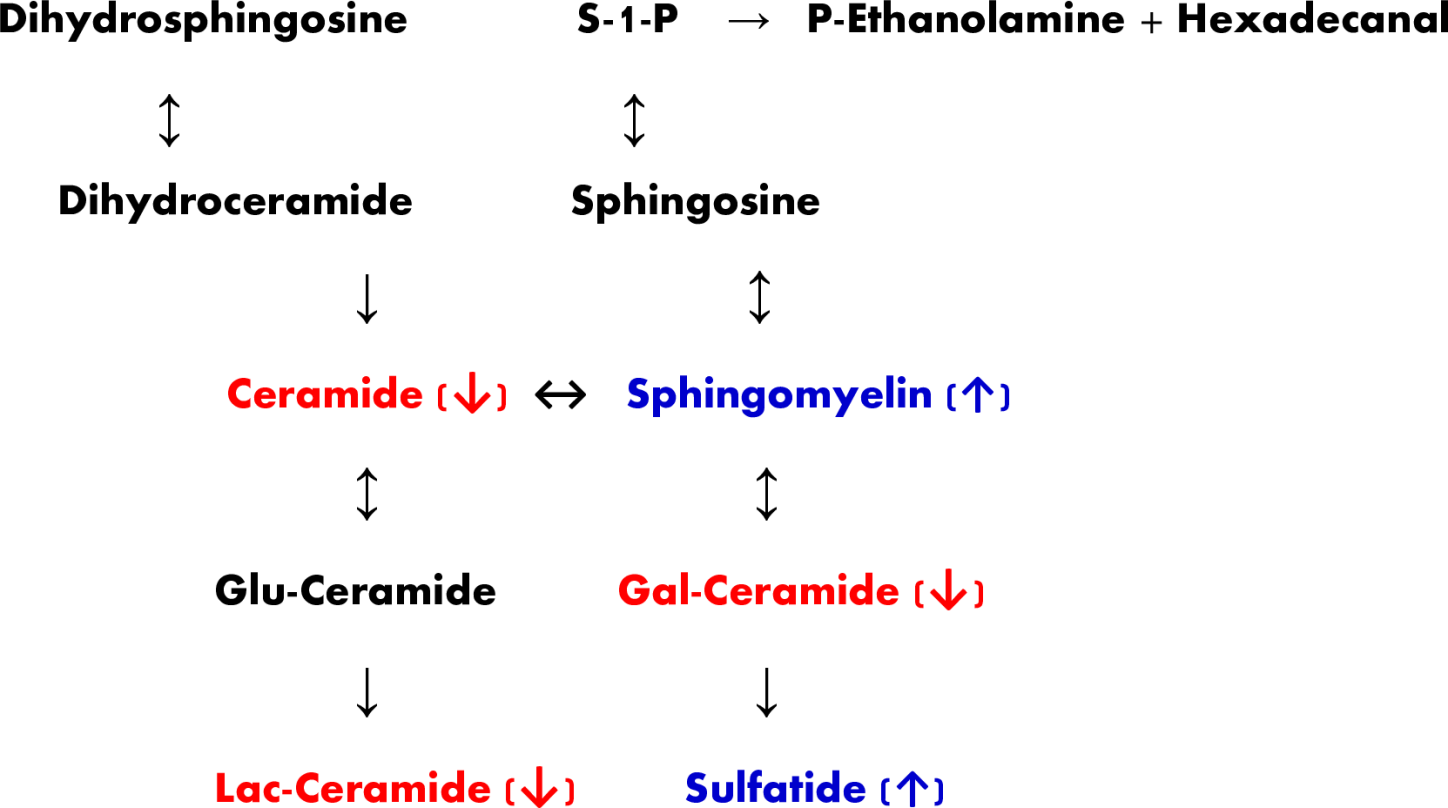
Abbreviated schematic presentation of sphingolipid metabolism. Gal, galactosyl; Glu, glucosyl; Lac, lactosyl; S-1-P, sphingosine-1-phosphate. Red indicates lowered levels and blue indicates increased levels observed in our study.

Recently, heterozygous mutations in glucocerebrosidase GBA1, which catalyzes the hydrolysis of glucosyl ceramides, have been shown to be the most common genetic risk factor for PD and LBD [54–57]. The underlying mechanism that produces this risk is unknown and both gain-of-function mechanisms involving abnormal GBA1 accumulation and loss-of-function mechanisms that purport decreased GBA1 activity have been hypothesized as mechanisms [58]. The lack of a consistent phenotype of PD or LBD in patients with GBA1 mutations, viewed as one argument against a loss-of-function mechanism, may simply indicate the complexity of multifactorial disease in which other factors interact with both GBA1 and alpha-synuclein to produce the pathologic findings and clinical symptoms of disease. Similarly, mutations in LRRK2, the chief cause of hereditary PD, have been modeled in mice and shown to increase ceramide levels in brain [59]. Glucocerebrosidase deficiency in PD and LBD would be expected to increase glucocerebroside content of brain tissue. Secondary effects of this might include increases in ceramide and its constituent sphingolipids, of which our current work demonstrates increases in both sphingomyelins and sulfatides that are not present in AD alone but are seen specifically when neocortical Lewy bodies are present. In addition, our non-biased analytical platform revealed decrements in cortical ceramide levels as previously reported [60]. We also detected decrements in galactosylceramide 24:1 and/or gluclosylceramide 24:1 in PD cortex, which cannot be differentiated by direct infusion lipidomics. However, previous studies have not detected alterations in cortical glucosylceramides in PD [61], therefore we assume the monitored decreases were in galactosylceramide (Fig 7). Large decrements in lactosylceramides were monitored in all 3 neuropathological groups further supporting complex alterations in sphingolipid metabolism in these disorders (Fig 7).

Previously, we determined plasmalogens, ether phospholipids that are abundant in brain tissue and hypothesized to protect against reactive oxygen species [61], to be decreased in AD [62–63]; our current study demonstrates this not to be the case, however, in cerebral cortex affected by PD and LBD. As LBD and PD feature less global cerebral cortical damage and atrophy than AD, this might be anticipated. However, surprisingly, we found plasmalogens and especially those containing ethanolamine are increased in PD and LBD cortex compared to even controls.

## Conclusions

Accumulation of DAGs in AD cortex appears to be a consistent finding among a number of lipidomics laboratories. We have extended these findings to include PD, LBD with intermediate AD pathology, and LBD with AD pathology. Considering the tight control of the homeostatic regulation of DAG levels and the multiple roles of these neutral lipids, continued research into their potential role of DAG dysfunction in neurodegenerative changes resulting in cognitive decline is imperative. In addition, it is imperative to determine if plasma DAG levels are elevated in PD and LBD subjects, as they are in MCI and AD patients. If this is the case plasma DAG levels may represent a useful biomarker for future neurodegeneration and thereby provide a rationale for early NSAID intervention to block or slow the disease processes invoked by neuroinflammation. Early intervention is critical to the success of any anti-inflammatory approach [52].

## Supporting information

S1 Table Individual R values for all presented data (R = ratio of endogenous lipid peak area to peak area of the internal standard). (XLSX)
